# Effects of bioturbation on environmental DNA migration through soil media

**DOI:** 10.1371/journal.pone.0196430

**Published:** 2018-04-24

**Authors:** Christopher M. Prosser, Bryan M. Hedgpeth

**Affiliations:** ExxonMobil Biomedical Sciences Incorporated, Annandale, NJ, United States of America; University of Hyogo, JAPAN

## Abstract

Extracting and identifying genetic material from environmental media (i.e. water and soil) presents a unique opportunity for researchers to assess biotic diversity and ecosystem health with increased speed and decreased cost as compared to traditional methods (e.g. trapping). The heterogeneity of soil mineralogy, spatial and temporal variations however present unique challenges to sampling and interpreting results. Specifically, fate/transport of genetic material in the terrestrial environment represents a substantial data gap. Here we investigate to what degree, benthic fauna transport genetic material through soil. Using the red worm (Eisenia fetida), we investigate how natural movement through artificial soil affect the transport of genetic material. All experiments were run in Frabill® Habitat® II worm systems with approximately 5 cm depth of artificial soil. We selected an “exotic” source of DNA not expected to be present in soil, zebrafish (Danio rerio) tissue. Experiment groups contained homogenized zebrafish tissue placed in a defined location combined with a varying number of worms (10, 30 or 50 worms per experimental group). Experimental groups comprised two controls and three treatment groups (representing different worm biomass) in triplicate. A total of 210 soil samples were randomly collected over the course of 15 days to investigate the degree of genetic transfer, and the rate of detection. Positive detections were identified in 14% - 38% of samples across treatment groups, with an overall detection rate of 25%. These findings highlight two important issues when utilizing environmental DNA for biologic assessments. First, benthic fauna are capable of redistributing genetic material through a soil matrix. Second, despite a defined sample container and abundance of worm biomass, as many as 86% of the samples were negative. This has substantial implications for researchers and managers who wish to interpret environmental DNA results from terrestrial systems. Studies such as these will aid in future study protocol design and sample collection methodology.

## Introduction

Accurate biodiversity assessments are a central component to compliance with environmental regulations. In the United States, for example, environmental impact statements under the National Environmental Policy Act require extensive baseline information on biodiversity. Similarly, quantitative biodiversity assessments are important for assessing the progress of habitat reclamation efforts. However, traditional biodiversity monitoring relies on direct (ex. traps, sightings) or indirect (ex. tracks, calls) observation of organisms. Especially true for direct methods such as trapping or netting, these activities are often time consuming, expensive, and impractical in remote or hard to reach regions.

Over the past decade, technological advances have resulted in the ability to detect the presence of organisms through amplification of environmental DNA (eDNA). eDNA is a generic term collectively referring to all genetic material that can be extracted from environmental media. Examples are extracellular DNA fragments, hair, feces, blood, free microbial cells, pollen or any other source by which cells and/or tissue may enter the environment [1]. Due to high precision, species-specific detection and low rates of false positives, eDNA has been increasingly utilized for an array of studies including biodiversity assessments, mapping of species distributions, and detection of invasive and endangered species [1–5]. DNA-based ecosystem monitoring can have distinct advantages over traditional sampling methods, including being less invasive/less destructive than trapping/netting. Sampling only environmental media (water, soil, sediment), reduces stress and danger of entrapment of valuable (e.g. endangered) species in nets or snares.

The DNA sequencing of bulk material containing the DNA of dozens or hundreds of species would have been cost-prohibitive with older low throughput DNA sequencing platforms (e.g. Sanger sequencing). However, with next generation DNA sequencers (NGS), which use high-throughput technologies such as massively parallel sequencing, it is now possible to generate millions of DNA reads from bulk material in a short period of time [6]. Additionally, newer DNA sequencing technologies boast low detection limits (10^−8^ ng/μL) allowing for low levels of genetic material to be amplified and sequenced.

To date, the majority of eDNA studies have focused on aquatic and/or wetland systems [3, 7–9]. This is most likely due to methodological advantages of sampling aquatic media. For example, lotic and lentic systems provide defined boundaries within which to sample and relatively large volumes of water (as large as several liters) can be filtered to concentrate available genetic material. In contrast to eDNA analysis from aquatic/marine systems, there is generally a paucity of data from terrestrial habitats. Soil matrices present unique challenges that are not encountered in aquatic systems. For example, the volume of soil used in extractions is typically a limiting factor (~0.25g soil per extraction). Additionally, little is known on eDNA fate and mobility in terrestrial systems over time and space (i.e. once deposited, there is little data to predict transport and/or persistence). A non-detect may be a false-negative if in fact the complexity of soil matrix precludes homogenous distribution of genetic material thus limiting spatial area from which it can be detected.

As compared to aqueous media, the chemical complexity and reactivity of soils displays a greater degree of spatial and temporal heterogeneity, raising questions about eDNA mobility in soils. Soil mineralogy (e.g. clay, sand, silt) and subsequent mixtures (e.g. silty clays) will greatly influence the amount of surface reactive particles present, and thus the adsorption of genetic material within that matrix [10]. Physicochemical interactions influencing eDNA mobility within the soil matrix are highly variable and will depend on DNA fragment size, soil mineralogy, hydrophobicity, pH and ionic strength [11]. Persistence of eDNA in soils has also received limited attention and is incompletely understood. The presence of clay and soil colloids has been suggested to prohibit enzymatic degradation of genetic material thus potentially prolonging its availability for detection [12,13]. In anoxic environments, such as lake sediment, eDNA has been recovered dating back thousands of years [14]. Conversely, eDNA can also be taken up by bacteria as a source of nutrition expediting its removal from the environment [10]. Such uncertainties have led to wide estimates in persistence ranging from days to years in the top 15 cm of soil [15].

To date, few field studies have been conducted specifically focused on eDNA extraction from soil. However, in recent years researchers have investigated soil samples from natural wetland habitats [16] as well as in more spatially defined zoos and parks [17]. Fahner et al. [16] investigated large-scale plant monitoring using DNA metabarcoding. Researchers collected core samples from the Ramsar designated Peace-Athabasca Delta in Wood Buffalo National Park, Alberta, Canada with the goal of identifying standard DNA markers designed to evaluate floral biodiversity. An important approach in this study was the targeting of full length amplicons (400–900 base pairs), demonstrating this length is not so extensively degraded to preclude their use in biodiversity assessment.

Andersen et al. [17] investigated a fundamental relationship between known species abundance and detectable levels of eDNA. Researchers isolated and amplified eDNA from known species in safari parks, zoological gardens, and farms and found that detectable eDNA generally reflected the diversity of animals on the landscape. However, these researchers reported patchy detection (as low as 31%) from soil surface. Researchers also reported eDNA extraction efficiency was inversely proportional to organic carbon content of the soil.

The vast majority of studies to date have focused on the presence/absence of DNA in the environment; however, such studies do little to investigate eDNA fate and transport. While there are some exceptions in aquatic systems (i.e. [8]), there is a noticeable data gap investigating such effects in terrestrial systems. While the deposition of genetic material through normal processes (e.g. hair loss) is generally accepted, the degree to which physical (e.g. wind/rain) and biological (bioturbation) processes disseminate genetic material through terrestrial media are not well understood. As eDNA continues to grow as a tool for use in ecological assessments, a fundamental understanding of detection rate, and the risk of false negatives in terrestrial media will bolster data interpretation.

Given the paucity of data related to eDNA fate and transport within terrestrial environments, the scope of this study focused on whether bioturbation will transport eDNA through soil. Our study was designed to investigate if normal biotic activity (e.g. the natural movement of worms through soil) would transport detectable levels of genetic material from a single, well defined depositional source, to adjacent areas. The redworm (*Eisenia fetida*) was used in controlled laboratory experiments to examine if, and to what degree bioturbation moves DNA from a single deposition source through soil.

## Materials and methods

### *Eisenia fetida* husbandry

*Eisenia fetida* are a common laboratory test species (e.g. OECD guidance documents 207 and 222) for which well-established husbandry methods have been developed and thus serve as an appropriate model species to investigate horizontal migration of genetic material through a soil matrix. Adult *E*. *fetida* (~3–4 inches long) from Carolina Biological Supply Company^®^ (Burlington, NC, USA) were cultured in Frabill^®^ HABITAT V^®^ long term storage systems. Worms were cultured according to OECD guideline 222. Magic^®^ worm bedding (sphagnum peat moss base), moistened with house-generated deionized water, was selected as the culture media (i.e. artificial soil). Cultures were fed Magic^®^ worm food *ad lib* 1–2 times weekly. General health was monitored weekly with any abnormalities noted and removed from culture.

### Test procedure

Zebrafish (*Danio rerio*) were selected as the novel source of genetic material (zDNA). This was done for two primary reasons; first, the zebrafish genome has been published and common primer sets are readily available. Second, the simplest way to evaluate eDNA migration is to use a foreign or novel source not expected to be present in the test media (i.e. artificial soil). Therefore, genetic material from a teleost in a terrestrial system served as the foreign DNA source. To ensure there were no false positives, zDNA primers were tested against worm tissue, worm food, virgin artificial soil (i.e. straight out of the package before worms added), and mature artificial soil (i.e. after worms were added and the culture was well established).

All experiments were run in Frabill^®^ Habitat^®^ II worm storage systems (39 x 18 x 27 cm) filled with approximately 5 cm artificial soil. (http://www.frabill.com/open-water-fishing/aeration-bait-care/worm-care/1020.html). A total of five treatment groups were analyzed in triplicate: 1) Negative control: artificial soil with no zDNA added and no worms, 2) eDNA control: artificial soil with zDNA added but no worms, 3) ten worms per container with zDNA added, 4) thirty worms per container with zDNA added and 5) fifty worms per container with zDNA added. All treatment groups were kept at room temperature (~23°C); lids on the worm storage systems resulted in near continuous darkness except during periods of soil sampling. The negative control and eDNA control were essential to ensuring there was no cross contamination when test systems were moved and/or lids opened and closed during sampling events. The eDNA control ensured that if any zDNA was detected during sampling events, it was solely from worm movement and not from accidental movement of the genetic material during normal maintenance/sampling.

**Fig 1:**
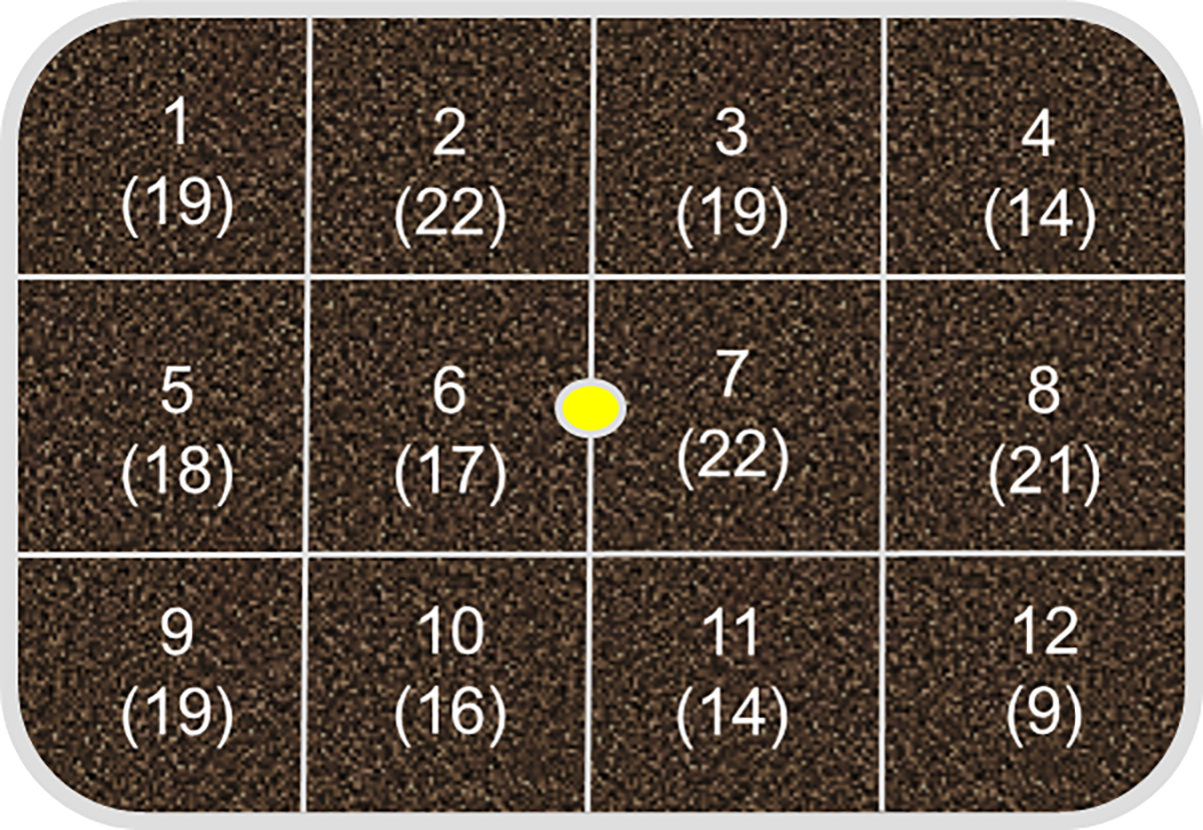
Diagram of sampling box with grid locations numbered. Parentheses numbers indicate total number of times the grid was sampled across all treatment groups and days. Yellow circle in the center denotes deposition location of zDNA.

For treatment groups receiving zDNA (groups 2–5), whole fish were homogenized directly into artificial soil and placed in the center on the container. First, adult zebrafish, previously sacrificed fish which had been stored at -20° C, were removed from storage and thawed. Fish were dried overnight in a VWR® Gravity Convection oven (model 414008–112) at 70°C. Dehydrated zebrafish were ground up by hand using a mortar and pestle and then further homogenized into artificial soil to create a mixture proportion of 100 mg zebrafish tissue:1 g artificial soil. A final weight of 10 g of zebrafish/artificial soil mixture was carefully added into a circular plot, extending through the depth of the soil column, in the center of the test vessel for groups 2–5 (Fig 1).

Each test container was gridded out into twelve equal sized sections (Fig 1). Laboratory observations of *E*. *fetida* indicate they move randomly throughout their enclosure ensuring an indiscriminate locations during each sample period. Therefore, a random number generator was used to select the grid sections from which a sample would be taken. Two sections were selected on each sampling day and approximately 1 g sample was taken from the middle of those sections. There were a total of seven sampling events over a fifteen day period resulting in a total of 210 soil samples being taken (42 total samples per treatment; 14 per replicate). Soil samples were taken with a 6.5 inch Lab Scoop Scoopula, this allowed for a verticle “core” sample to be collected. Multiple sterile scoopulas were used to prevent cross-contamination. Scoopulas were rinsed with 70% ethanol, Cole-Parmer DNA surface decontaminant and deionized water prior to soil sampling. Approximately 1 g of soil samples was placed into 2 mL Eppendorf tubes and kept frozen (-20°C) until extraction. All extractions were performed using ~ 0.2 g of soil with a PowerSoil® DNA Isolation Kit (Mo Bio Laboratories Inc.) according to manufacturer’s instructions. Following extraction, NanoDrop™ 2000c was utilized to identify samples which contained high quality DNA. Upon DNA verification, extractions were kept frozen (-20°C) until PCR amplification. PCR amplification was done on all samples from the eDNA and negative controls, but only on samples from the treatment groups from which the NanoDrop™ 2000c verified high quality DNA (S1 Table).

### PCR amplification and gel electrophoresis

Zebrafish primers previously reported in literature were used for amplification [18]. Porphobilinogen deaminase [PBGD; 695 bp] (F: TCTGGAGGACTGTAAGAGGTATGC; R: AGACGCACAATCTTGAGAGCAG) was selected as the target gene due to its base pair length for easy detection using gel electrophoresis and exotic nature (i.e. not expected to be in worm tissue, food, or bedding). Primers were purchased from Integrated DNA Technologies Inc. (http://www.idtdna.com), reconstituted according to manufacturer’s instructions and stored at -80°C until use. To ensure there were no false positives, zebrafish primers were tested against worm tissue, worm food, virgin worm bedding (i.e. straight out of the box before worms were added), and mature worm bedding (i.e. bedding that had worms living in it). PCR was carried out in a Veriti® 96-Well Thermal Cycler with a total 50 μL final volume. Each well contained 45 μL Platinum® PCR SuperMix (Invitrogen), 3 μL DNA template and 1 μL each of the forward and reverse primers. PCR was performed under conditions recommended by Platinum® PCR Super protocol: 30 cycles were run denaturing at 95°C for 15 seconds, annealing at 55°C for 30 seconds and final extension at 72°C for one minute per kb. ThermoFisher Scientific^®^ E-gel precast agarose electrophoresis system was used to observe DNA within target length. Banding size was estimated by comparison to TrackIt™ 100 bp DNA Ladder with a maximum band at 2072 bp.

## Results

DNA primer pairs were tested against both wet and dried zebrafish tissue. This was done to ensure the overnight drying process did not degrade zDNA. Positive detection was defined as an observable band after gel electrophoresis matching the expected base pair length of the amplicon of the target gene. The PBGD primers successfully amplified zDNA from both wet and dry tissue, resulting in an amplicon of the correct size. There were no amplifications from any of the non-zDNA sources (e.g. worm food/bedding) indicating that no false positives occurred (data not shown).

**Fig 2:**
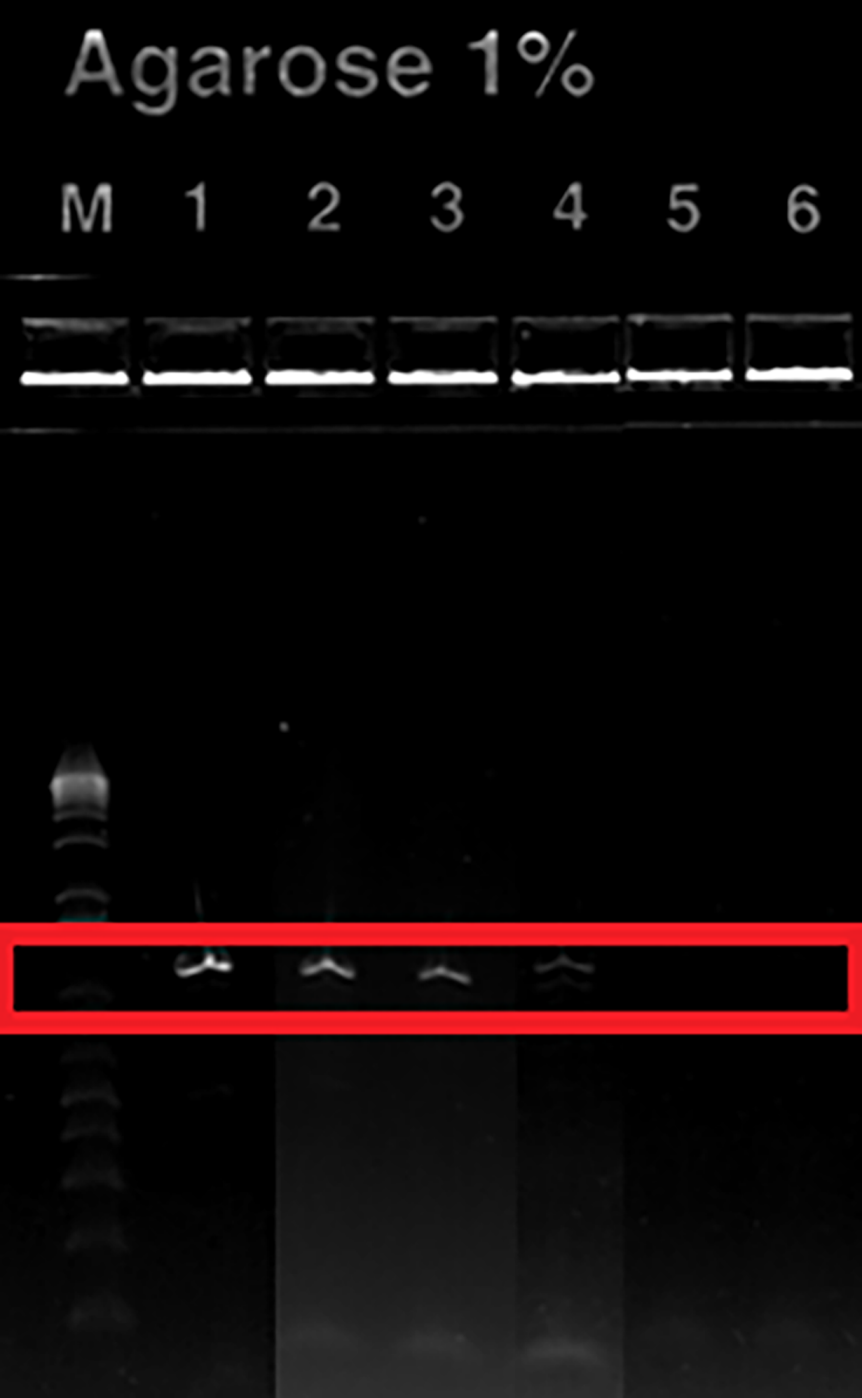
Banding of PBGD amplicons showing positive detection of zDNA isolate from the soil matrix. **(Image is a compilation of multiple gels)**. Lane M: ladder; Lane 1: dehydrated zebrafish tissue; Lane 2: 10 worm/replicate treatment; Lane 3: 30 worm/replicate treatment; Lane 4: 50 worm/replicate treatment; Lane 5: Negative control; Lane 6: eDNA Control.

To ensure there was no accidental or unintentional zDNA contamination, two control groups were used. A negative control contained neither worms nor zDNA. This group was used to ensure the opening and closing of lids, incidental contamination of scoopulas and/or other unforeseen events could contaminate soil. An eDNA control contained the central deposition site of zDNA, but no worms. This was to ensure lid opening/closing or movement of the boxes would not inadvertently move zDNA from the centralized spot to the sample grids. Using PBGD primers, no zDNA was amplified from soil samples in either the positive or negative controls (Fig 2). This suggests there was no contamination from sampling procedure or accidental transport of genetic material into control containers from those containing zDNA. This lends confidence that those samples from which zDNA were amplified did result from bioturbation of the genetic material and not accidental contamination.

A random number generator was used to determine from which grid locations soil would be taken. Over the duration of the experiment, sampling was distributed randomly throughout each treatment group, with every grid section being sampled at a minimum of nine times (Table 1, Fig 1). Total number of samples taken from each grid section varied from 9 (grid #12) to 22 (grids # 2 and #7); number of samples per grid varied due to the randomization of sampling location. Positive detections were observed in all three treatment groups that had worms (Table 1, Fig 2). The only grid without a positive detection was #10. Detections were spatially distributed randomly throughout the sample grid without positive detections clustered into a specific area or region of the box (Table 2 provides a representative example for the 10 worm/replicate group; other test groups are not shown).

**Table 1:** Total samples taken (for all replicates) per grid location further delineated by the number of zebrafish PBGD detections.

Grid number	Total samples per grid	PBGD detects (Negative control)	PBGD detects (eDNA control)	PBGD detects (10 worms/ rep)	PBGD detects (30 worms/ rep)	PBGD detects (50 worms/ rep)	Total PBGD detects
**1**	19	0	0	3	2	1	6
**2**	22	0	0	1	1	0	2
**3**	19	0	0	1	2	0	3
**4**	14	0	0	0	1	0	1
**5**	18	0	0	2	2	0	4
**6**	17	0	0	0	1	0	1
**7**	22	0	0	0	2	2	4
**8**	21	0	0	0	2	2	4
**9**	19	0	0	2	1	0	3
**10**	16	0	0	0	0	0	0
**11**	14	0	0	0	2	0	2
**12**	9	0	0	1	0	1	2
**Total**	**210**	**0**	**0**	**10**	**16**	**6**	**32**
% PBGD^*^		**0**	**0**	**24%**	**38%**	**14%**	**25%**

* %PBGD was calculated by dividing the number of positive detections by the total number of samples for each treatment. E.g. for 10 worms per rep: 24% = 10 positive detections ÷ (210 total samples ÷ 5 treatment groups) * 100

**Table 2:** Random distribution of sampling and detections across time and space for 10 worm/replicate group.

	Sample Time (d)						
Sample location	1	2	3	4	5	6	7
**1**	2(+)(-)	1(+)		1(+)		1(-)	
**2**	1(+)	1(-)				1(-)	
**3**				1(+)		1(-)	1(-)
**4**	1(-)			1(-)	2(-)		1(-)
**5**		1(-)	1(+)	1(-)			1(+)
**6**		1(-)	1(-)				
**7**					1(-)		1(-)
**8**			2(-)		1(-)		1(-)
**9**	1(+)		2(-)(+)	1(-)	1(-)		1(-)
**10**	1(-)	1(-)				2(-)	
**11**		1(-)				1(-)	
**12**				1(+)	1(-)		

Numbers indicate number of times each location was sampled. (+) indicate sample was positive for zPBGD; (-) signs indicate sample was negative for zPBGD.

Differences were observed in the number of positive detections between the three treatment groups. Of the 42 samples per treatment group over the seven sample events, there were 10 (24%), 16 (38%) and 6 (14%) positive detections for the 10 worms, 30 worms and 50 worms exposure groups respectively. This resulted in a significant difference observed between the 30 and 50 worm groups (Students T-test; p = 0.008); however significant differences were not observed between any other groups. Across all three treatment groups positive detections were 25.4% (32/126).

**Fig 3:**
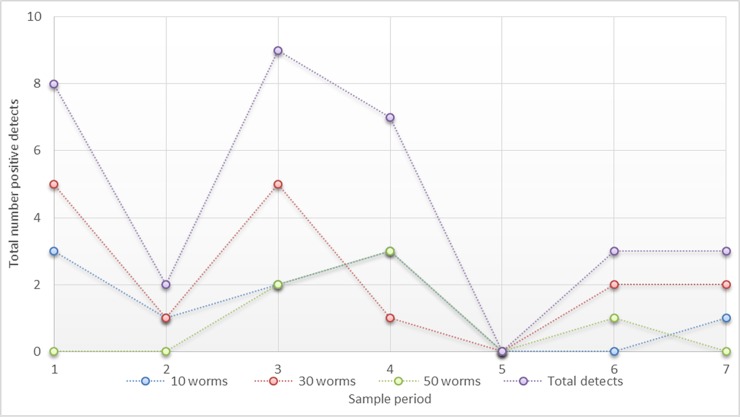
Temporal breakdown of positive zPBGD detections for each sampling event. Data are provided for each biomass level (sum of all three replicates) and total number of samples summed across all biomass levels. Total number of positive zDNA detections from sample events 1–4 is significantly higher than sampling events 5–7 (p = 0.02).

Temporal variations in positive detections were also evaluated using Students T-test. Fig 3 shows the breakdown of positive detections for each biomass level (summed across replicates) for each temporal sample event. All but sample event 5 had positive detections in at least one treatment. As seen in Fig 3, for all three treatment groups combined, there was a significant difference in the total number of positive detection in sampling events 1–4 versus 5–7 (p = 0.01). As noted above however, there was a significant difference in total detections between the 30-worm groups and 50-worm groups. To ensure the significantly lower number of detects in the 50-worm groups did not artificially deflate the temporal analysis, data was re-analyzed excluding the 50-worm group. Similarly, when only the 10-worm group and 30-worm group were combined, there still existed a significant difference in positive detections across sampling events 1–4 versus sampling events 5–7 (p = 0.02).

## Discussion

The purpose of this study was to enhance understanding of eDNA fate and transport in soil media. There is still a paucity of data related to spatial and temporal variations in eDNA concentrations as radial distance from a deposition site increases. Specifically, the effects of bioturbation on eDNA transport through soil media is wholly unknown. As such, researchers should be cognizant of the possibility of false negative occurrences. While these issues can be minimized with robust sampling design and high number of samples, a better understanding of eDNA fate/transport in soil media is essential for developing terrestrial sample methodology.

To our knowledge, this is the first controlled laboratory experiment designed to investigate the effects of bioturbation on eDNA migration in soil. Enhanced understanding of eDNA fate, transport and persistence in the environment is needed to allow for future utility in environmental monitoring applications. Results from this study demonstrate that the redworm (*Eisenia fetida*), and presumably other soil invertebrates, is capable of transporting genetic material from a centralized deposition location. This is clearly shown by the positive detects through the duration of the study for all three biomasses selected. The overall rate of detection for all three biomass treatments combined was 25.4%. Additionally, the high prevalence of positive detections in the box corners, demonstrates zDNA migration was only limited by the confines of the enclosure.

Despite the positive detections of zDNA throughout the enclosures, there were several observations that required further discussion. First, it was presumed that a higher biomass of worms would result in a higher prevalence of positive detections. While this is true between the 10 worm and 30 worm treatments, there were fewer positive detections in the 50 worm treatment. We speculate this is due to overcrowding of the worms. Previous experiments [19] have demonstrated crowding effects limiting growth and movement of *Eisenia fetida* over a twelve-week study. While we did not monitor growth of the organisms, crowding induced behavioral changes may have resulted in diminished movement within the container and thus less zDNA transport.

Conversely, the fewer positive detections may be accredited to zDNA degradation. As worms migrated through and consumed the feed / zDNA mixture, zDNA may have been degraded as it passed through the gut. This hypothesis however is not supported by looking at the 10 worm versus 30 worm treatment. If degradation due to passage through the gut were to explain the observations, we would have expected to see lower numbers of detections in the 30 worm treatment group. While the two groups were not significantly different (p = 0.1), the 30 worm treatment group did have more zDNA positive detections. The limitations of this study however, make it impossible to definitively conclude if, and to what effect digestive processes influenced eDNA degradation. Further work is required to fully explore if either of these hypotheses explain the observed differences in treatment groups.

A second observation dealt with the persistence of genetic material throughout the seven sampling periods. The first four sampling periods had a significantly higher number of positive detections than did the last three (p = 0.02). There are several possible reasons for this observation. The perceived decrease in positive detections over time may be due to natural causes (e.g. degradation through the gut) or may be an artifact of the migration. As worms transport zDNA away from the center, the random sampling from the edges of the container may disproportionately represent areas in which genetic material is more prevalent. However, evaluating sample locations temporally revealed that sampling bias was not likely to influence the results. Ultimately, this study was designed to investigate spatial changes and not temporal variations in zDNA detection. The significant findings may therefore be an artifact of sample design and statistical power precluding definite conclusions from being made. Future work should be designed with the appropriate methodology in place to further investigate these observations.

## Conclusions

The utilization of eDNA for field-based biodiversity assessments is growing rapidly. While aquatic systems have received most of the attention, knowledge of terrestrial habitats is plagued by a paucity of data. The goal of this work was to evaluate if, and to what extent, fate and transport of genetic material is impacted by bioturbation. It has been definitively shown that *Eisenia fetida* in soil media are capable of transporting genetic material spatially from a single point of surface deposition. It has also been shown that while zDNA was amplified throughout the duration of the experiment, positive detections were significantly reduced after the first week suggesting degradation of the genetic material. Unfortunately, limitations of this study cannot disentangle whether or not the degradation was from natural breakdown, or due to passing through the gut of the worm. From a practical point of view however, this may not matter to the researcher. Whether from physical/chemical process, or through digestion from the biota, it is important to understand the potential rates of eDNA detection and knowing that biotubation is a legitimate form of eDNA transport through soil media.

As researchers and scientists continue to explore new areas in which eDNA data may inform decision making, it is important to appreciate the challenges and limitations of such work. Here we have taken a first step to demonstrate that bioturbation will influence eDNA migration through soil. While this is only one of many factors that may impact the fate and transport of genetic material through soil, we demonstrated relatively low levels of detection (~25%). As such, researchers need to be cognizant of all the factors that may impact eDNA migration, realize the possibility for false negatives, and create robust field sampling methods to minimize their occurrence.

Future work will be aimed at gaining a better understanding of fate/ transport under actual environmental conditions. It is still wholly unknown how wind, rain and solar radiation will influence transport and persistence of a centralized deposition location of genetic material in soil. Additionally, by utilizing a non-ingestible form of genetic material, future research may begin to separate causes of eDNA degradation. As stated above, limitations of this study prohibit the differentiation of physical/chemical degradation or degradation due to digestion after consumption by the worms.

## Supporting information

S1 Table Supporting information containing data for extracted samples including name, date of extraction and nanodrop results. (XLSX)
